# Supplementary material for: The interactome of intact mitochondria by cross-linking mass spectrometry provides evidence for coexisting respiratory supercomplexes
Source: Mol Cell Proteomics. 2017 Dec 8;17(2):216–32. doi: 10.1074/mcp.RA117.000470 (PMC5795388; doi:10.1074/mcp.RA117.000470)
Supplement: Supplemental Data [file supp_RA117.000470_133922_0_supp_23978_fzffwf.zip › spectra_annotation/mito_DR_spectra_annotation/22-1-3-1-22-1.pdf]

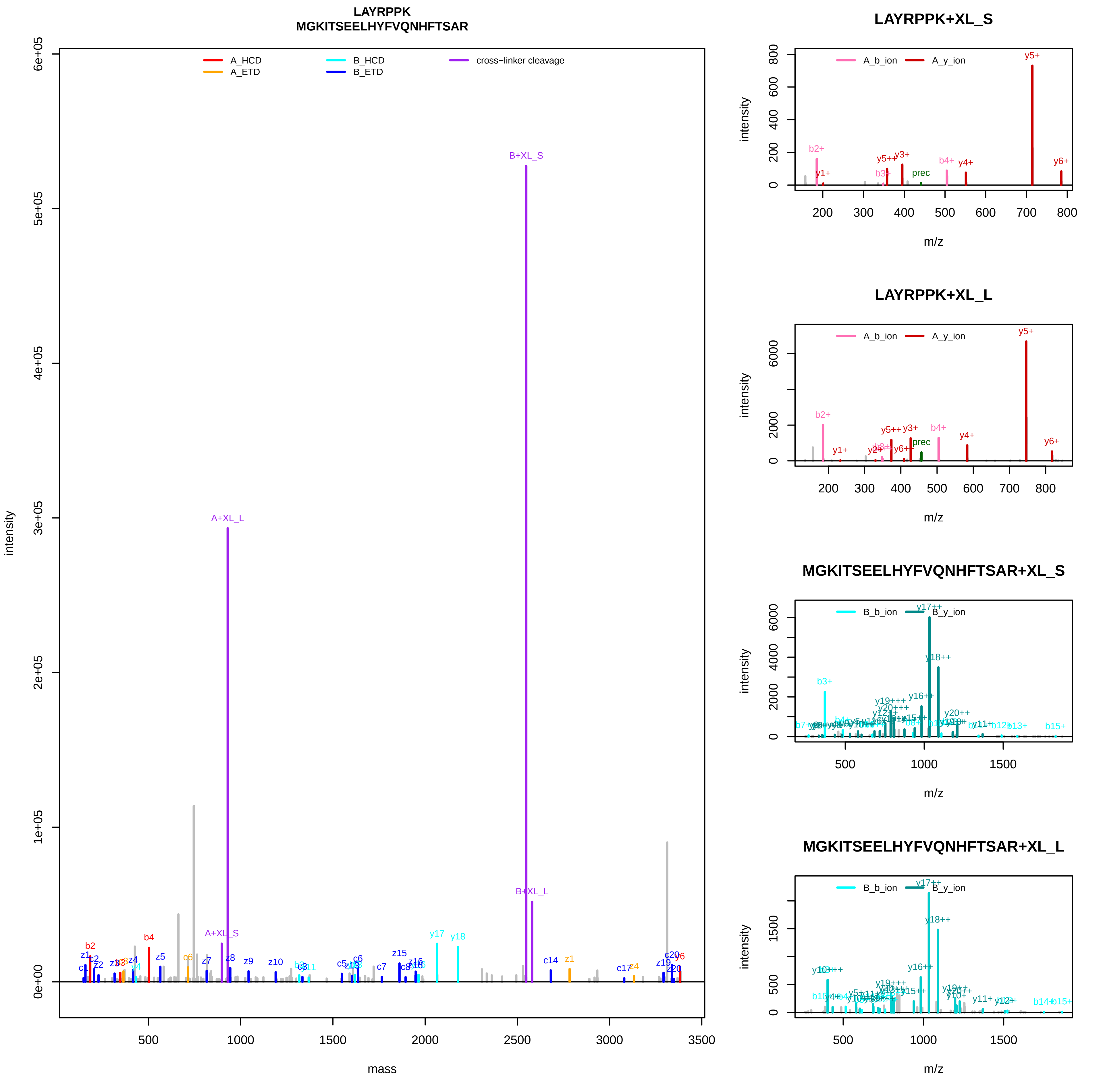

LAYRPPK
MGKITSEELHYFVQNHFTSAR
A_HCD
A_ETD
B_HCD
B_ETD
cross−linker cleavage
B+XL_S
A+XL_L
B+XL_L
A+XL_S
intensity
mass
0e+00
1e+05
2e+05
3e+05
4e+05
5e+05
6e+05
500
1000
1500
2000
2500
3000
3500
b2
b4
y6
y17
y18
z5
z7
z8
z9
z10
z15
c14
z1
c17
z19
c20
z20
LAYRPPK+XL_S
A_b_ion
A_y_ion
y5+
b2+
y5++
y3+
b4+
y4+
y6+
y1+
prec
m/z
intensity
LAYRPPK+XL_L
MGKITSEELHYFVQNHFTSAR+XL_S
B_b_ion
B_y_ion
y17++
y18++
b3+
y16++
y20++
b15+
MGKITSEELHYFVQNHFTSAR+XL_L
y10+++
b14+
